# Author Correction: Amazonian forest-savanna bistability and human impact

**DOI:** 10.1038/ncomms16179

**Published:** 2018-02-21

**Authors:** Bert Wuyts, Alan R. Champneys, Joanna I. House

Nature Communications 8: Article number: 15519; DOI: 10.1038/ncomms15519 (2017); Published: 05 30 2017; Updated: 02 21 2018

In the originally published version of this Article, reference 23 did not refer to the correct paper. This error has now been corrected in both the HTML and PDF versions of the Article.

