# Supplementary material for: Amazonian forest-savanna bistability and human impact
Source: Nat Commun. 2017 May 30;8:15519. doi: 10.1038/ncomms15519 (PMC5459990; doi:10.1038/ncomms15519)
Supplement: Supplementary Information — Supplementary Tables, Supplementary Figures, and Supplementary References [file ncomms15519-s3.pdf]

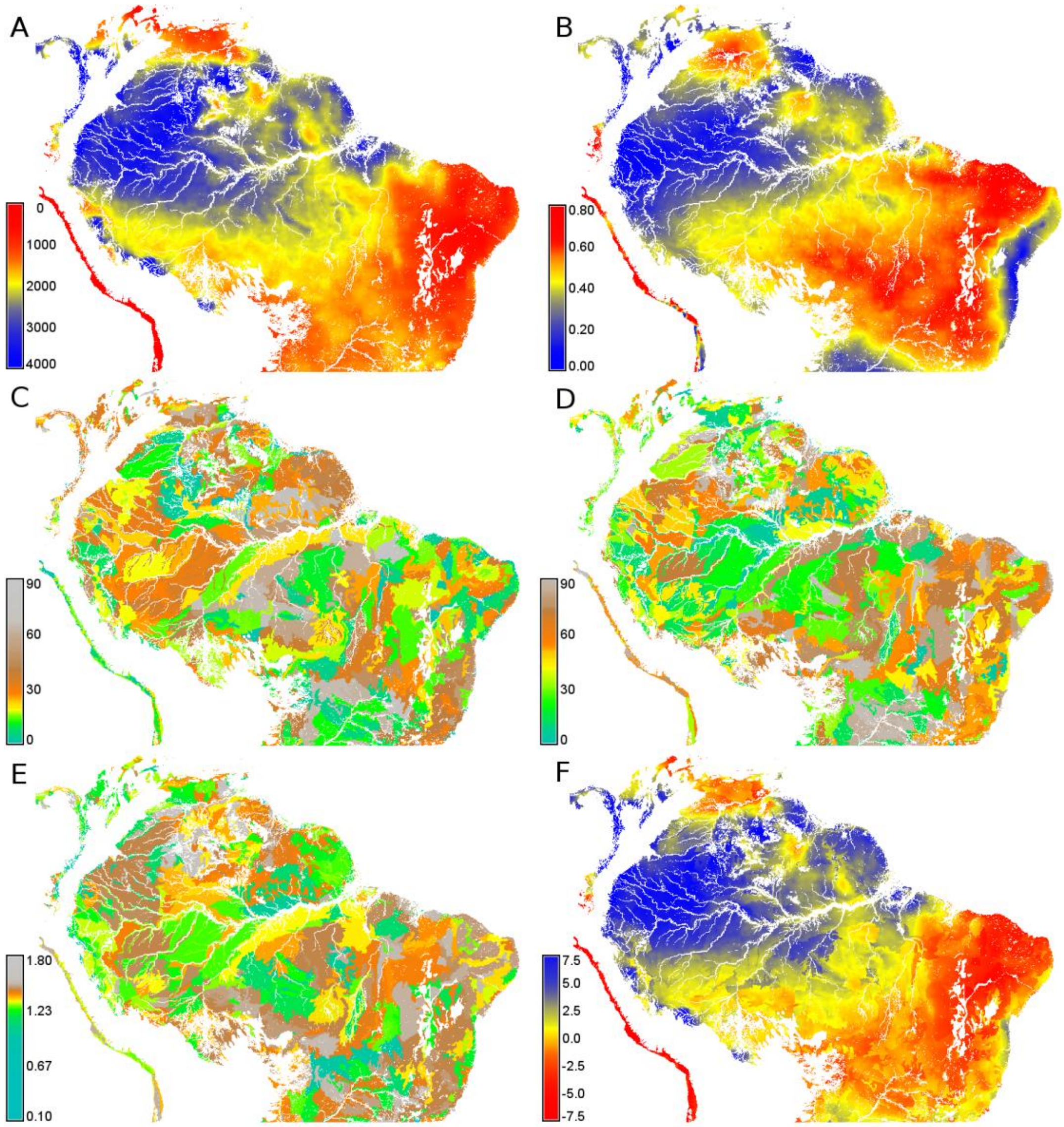

Supplementary Figure 1

Used variables for forest prediction and modelling. (A) Mean annual rainfall (MAR, in mm), (B) Markham's seasonality index (MSI), (C) Topsoil clay fraction (%), (D) Topsoil sand fraction (%), (E) Topsoil bulk density (kg m^{-3}), (F) Climatic-edaphic forest suitability index, obtained by a linear combination of climatic variables A,B plus and a non-linear combination of soil variables C-E.

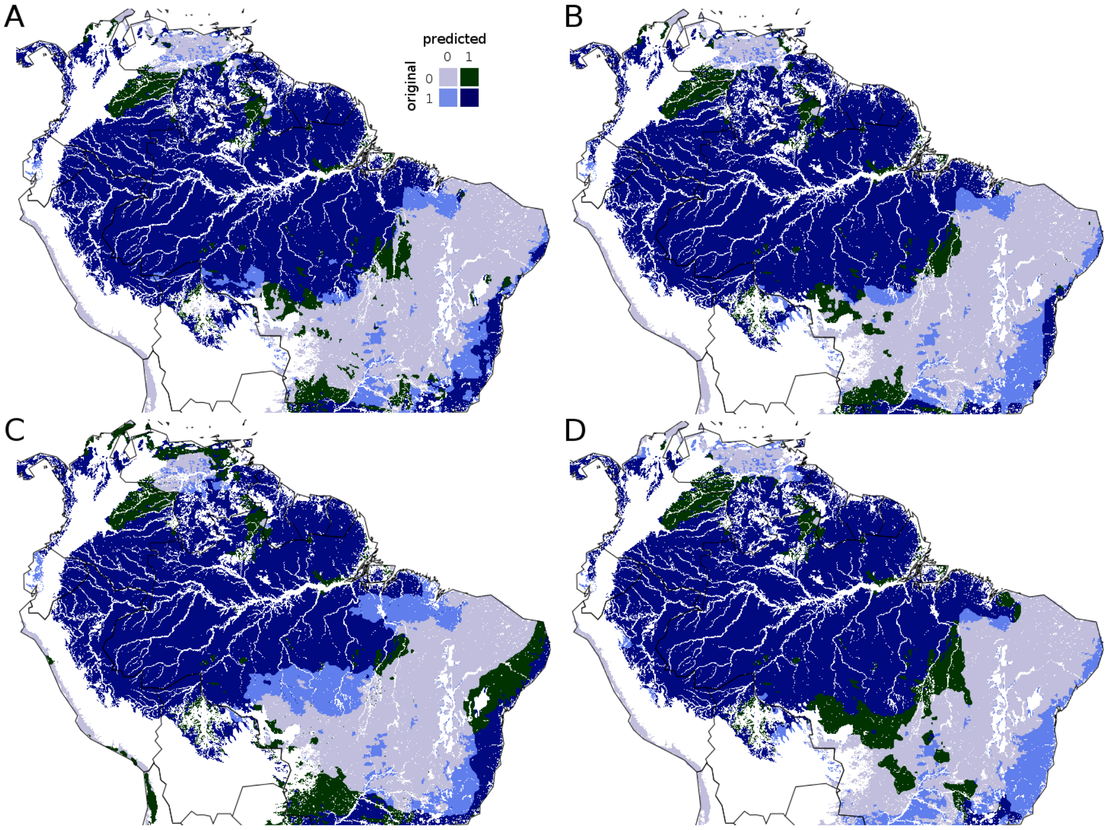

Supplementary Figure 2

Alternative prediction models: spatial prediction. Predicted original forest cover based on the logistic regression model of original forest cover as a function of: (A) mean annual rainfall (MAR), Markham seasonality index (MSI) and soils (same as Figure 1B), (B) MAR and MSI, (C) MSI only, (D) MAR only.

Supplementary Table 1. Alternative prediction models: model parameters.

i	θ_i	soil, MSI, MAR	MSI, MAR	MSI	MAR
0	Intercept	4.16	-1.18	5.13	-4.80
1	sand	2.38 e-02	-	-	-
2	clay	-1.88 e-01	-	-	-
3	density	-5.99	-	-	-
4	clay:density	1.83 e-01	-	-	-
5	MSI	-7.05	-6.29	-10.1	-
6	MAR	2.90 e-03	2.6 e-03	-	3.0 e-03
	Cohen Kappa	0.688	0.668	0.528	0.598

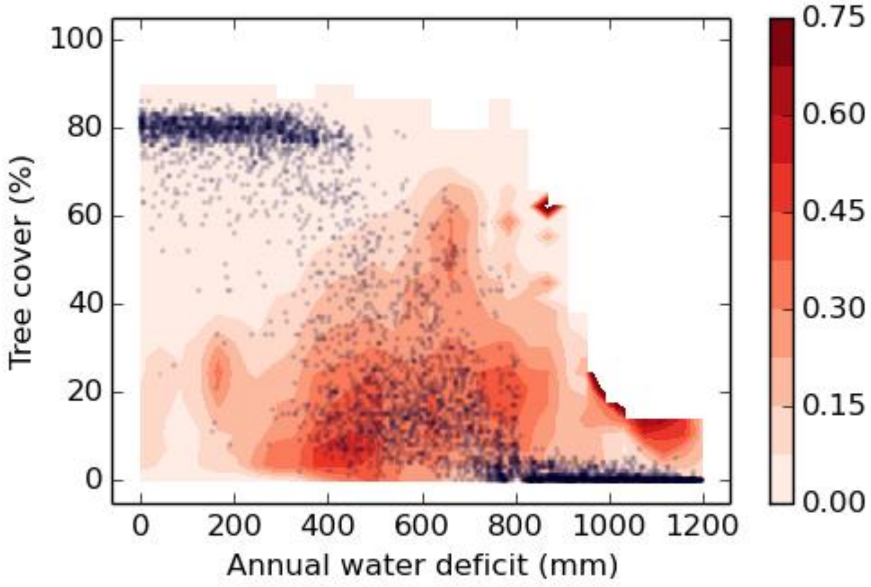

Supplementary Figure 3

Fire occurrence from the AQM burned area[1] data, MODIS VCF tree cover data[2] and TRMM[3]-based annual water deficits between 2005 and 2014. The colour scale indicates the fraction of areas that have experienced fire between 2005 and 2014 as a function of AWD and tree cover. This image was obtained by adding yearly data to their respective bins in (AWD;T_o) space, where AWD is mean annual water deficit and T_o observed tree cover in areas far from human impact. AWD contains the same information as CWD (r=0.99), climatic water deficit, which is used in many previous studies. AWD is easier to calculate. We assumed, as in previous work[4], an evapotranspirative demand in tropical rainforest of 100mm each month. AWD is the yearly sum of monthly shortages, or $AWD(y) = \sum_{m=1}^{12} \max(0, 100 - P_{y,m})$, where $P_{y,m}$ represents rainfall in month m of year y. Places without rain have a AWD of 1200mm (=12x100mm) and places where rainfall is never below 100mm have a AWD of 0mm. The scatterplot shows 2010 tree cover versus the mean of AWD over 2000-2010.

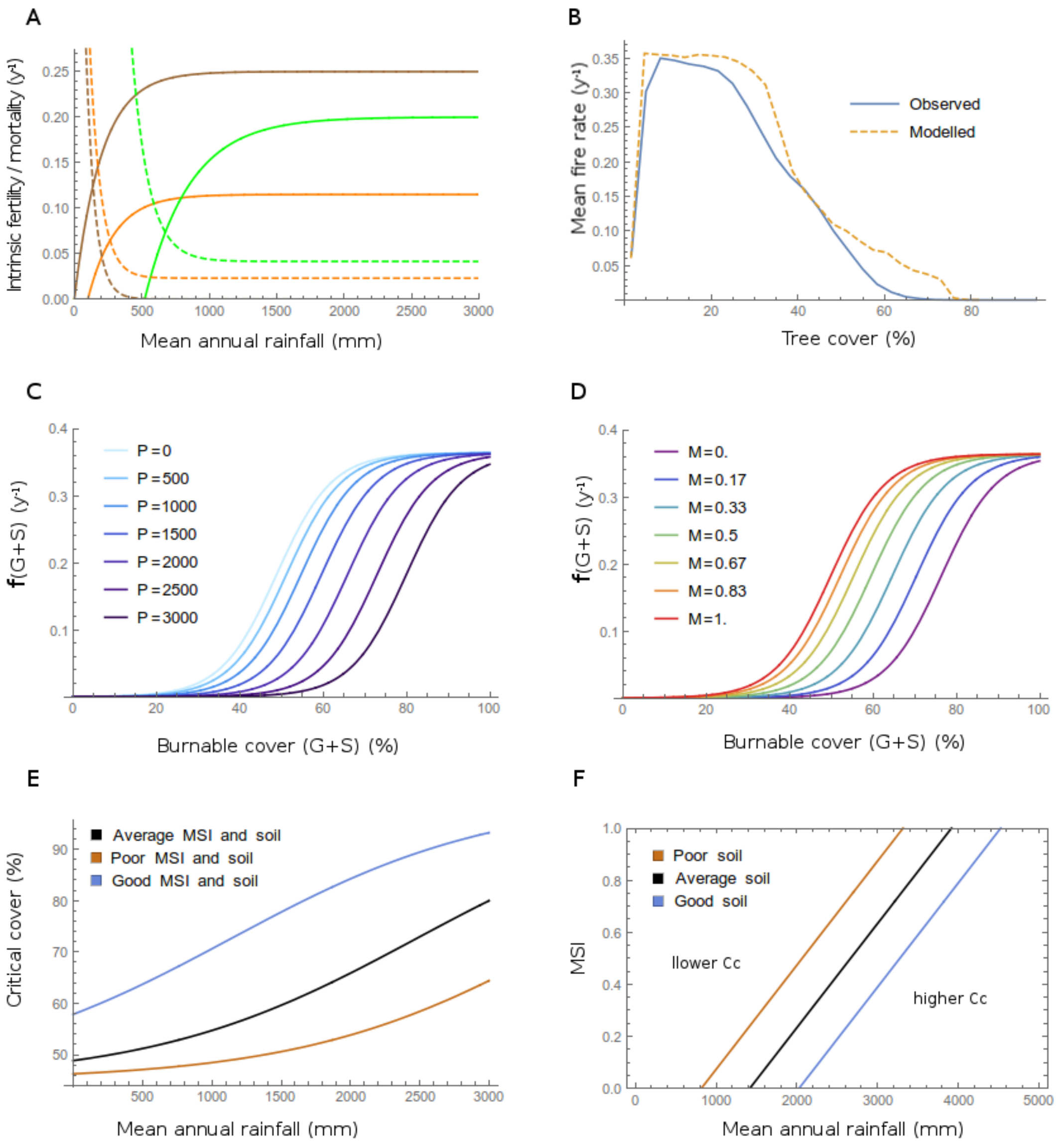

Supplementary Figure 4

Plots of model functions. (A) Growth (solid) and mortality rate (dashed) functions used in the model of forest *F* (green), savanna sapling/tree *S*/*T* (orange) and grass *G* (brown) cover types as a function of mean annual rainfall and for average soil and seasonality. Seasonality and soil sand fraction affects these functions by shifting them horizontally. (B) Mean fire rate derived from 10 model years as a function of tree cover *T*+*F* (dashed orange), compared to observed mean fire rate based on the AQM data set (2005-2014)[1] (C-D) Expected local fire rate function as a function of burnable cover *G*+*S* with the effect of rainfall (C) and seasonality (D) on critical burnable cover C_c at which fire occurrence rises sharply. (E) C_c as a function of mean annual rainfall, with the effects of seasonality and soils indicated. (F) Fire regimes occurring at different cover values separated in MAR,MSI space.

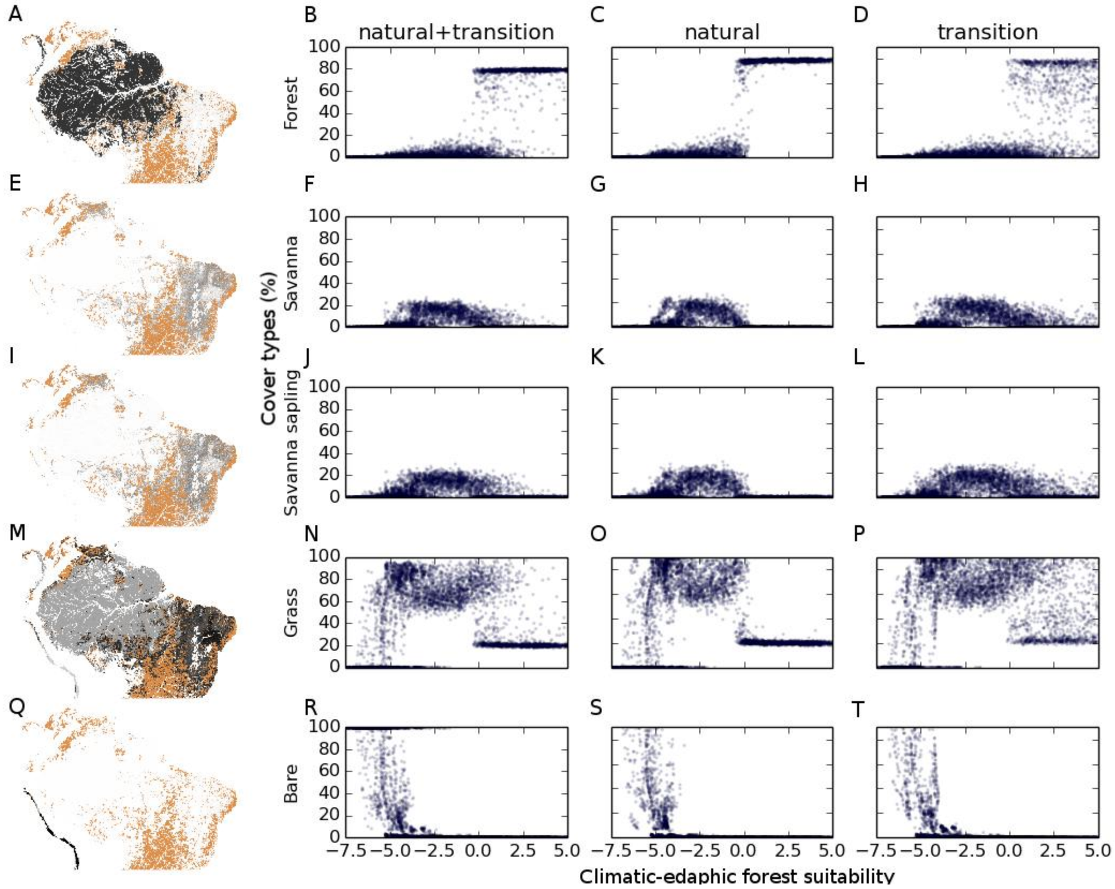

Supplementary Figure 5

Model results depicted similarly to Figure 5, differentiated by cover type: (A-D) Forest, (E-H) Savanna, (I-L) Savanna sapling, (M-P) Grass, (Q-T) Bare soil. The driest areas (CEFS<-5) consist almost exclusively of bare soil (Q). (C,G,K,O,S) In the natural zone, areas with $-5 \leq \mathrm{CEFS} \leq 0$ are affected by fires and drought. Hence, grasses, savanna saplings and savanna adult trees thrive here, with maximal cover of around 30% for savanna trees, 30% savanna saplings and 40 to 100% grass cover. Around $\mathrm{CEFS} \approx -2.5$, young and adult savanna trees reach their joint maximal cover of about 60%, with the remainder covered by grasses. Above $\mathrm{CEFS} \approx 0$, fire is infrequent enough for forest trees to be more competitive. (D,H,L,P,T) In the transition zone, portions of forests are converted to areas with low or zero forest tree cover and further colonised by a mix of grasses and savanna trees/saplings.

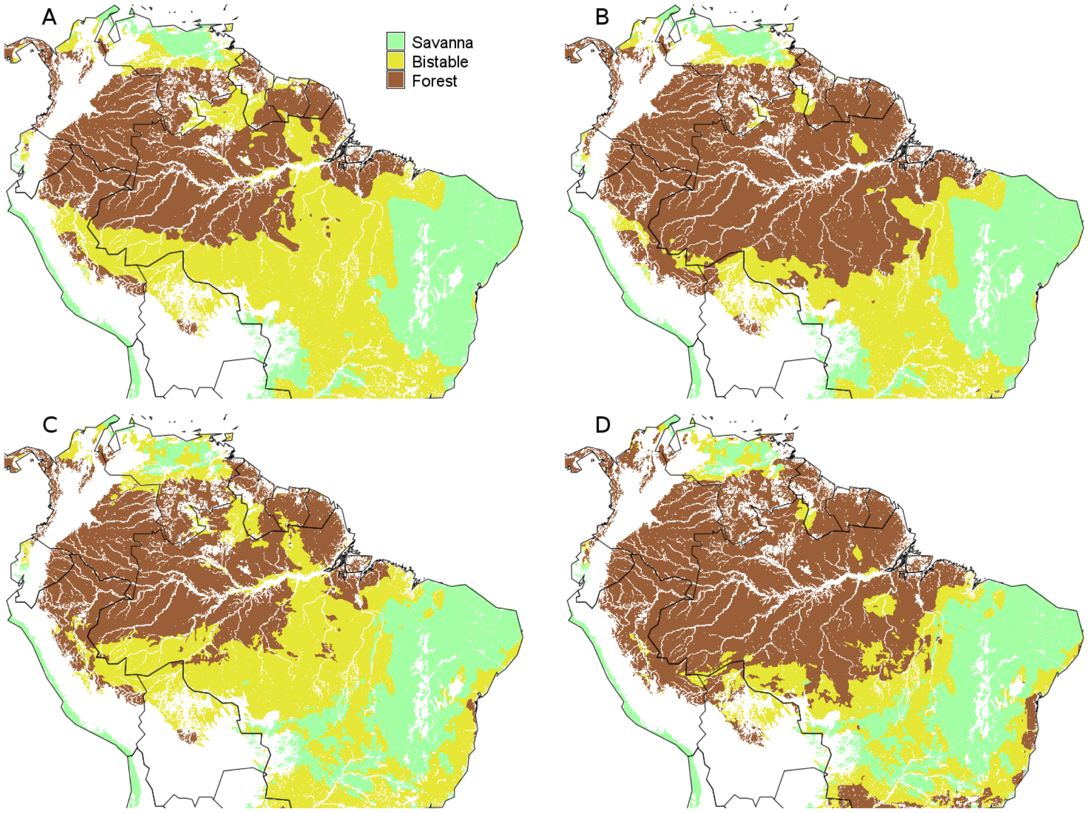

Supplementary Figure 6

Areas of stability inferred from the bimodality range in scatterplots of tree cover versus: (A) MAR in the whole study area except agriculutral areas, (B) MAR in natural areas, (C) CEFS in the whole study area except agriculutral areas, (D) CEFS in natural areas.Compare A,C to B,D to see the influence of anthropogenic edge effects and A,B to C,D to see the influence of accounting for natural spatial heterogeneity on the derived regions of stability.

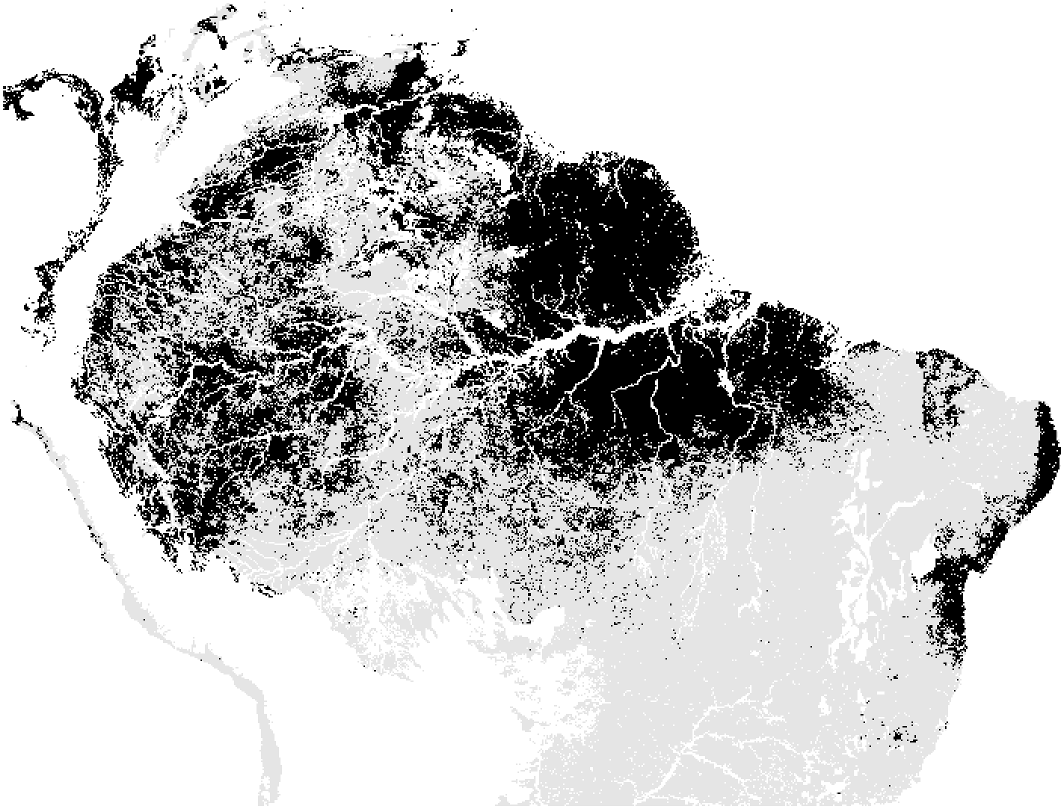

Supplementary Figure 7

MODIS VCF data quality. Areas where at least three out of seven periods used for reconstruction of tree cover in 2000 or in 2010 were affected by cloudiness or satellite-related issues (black).

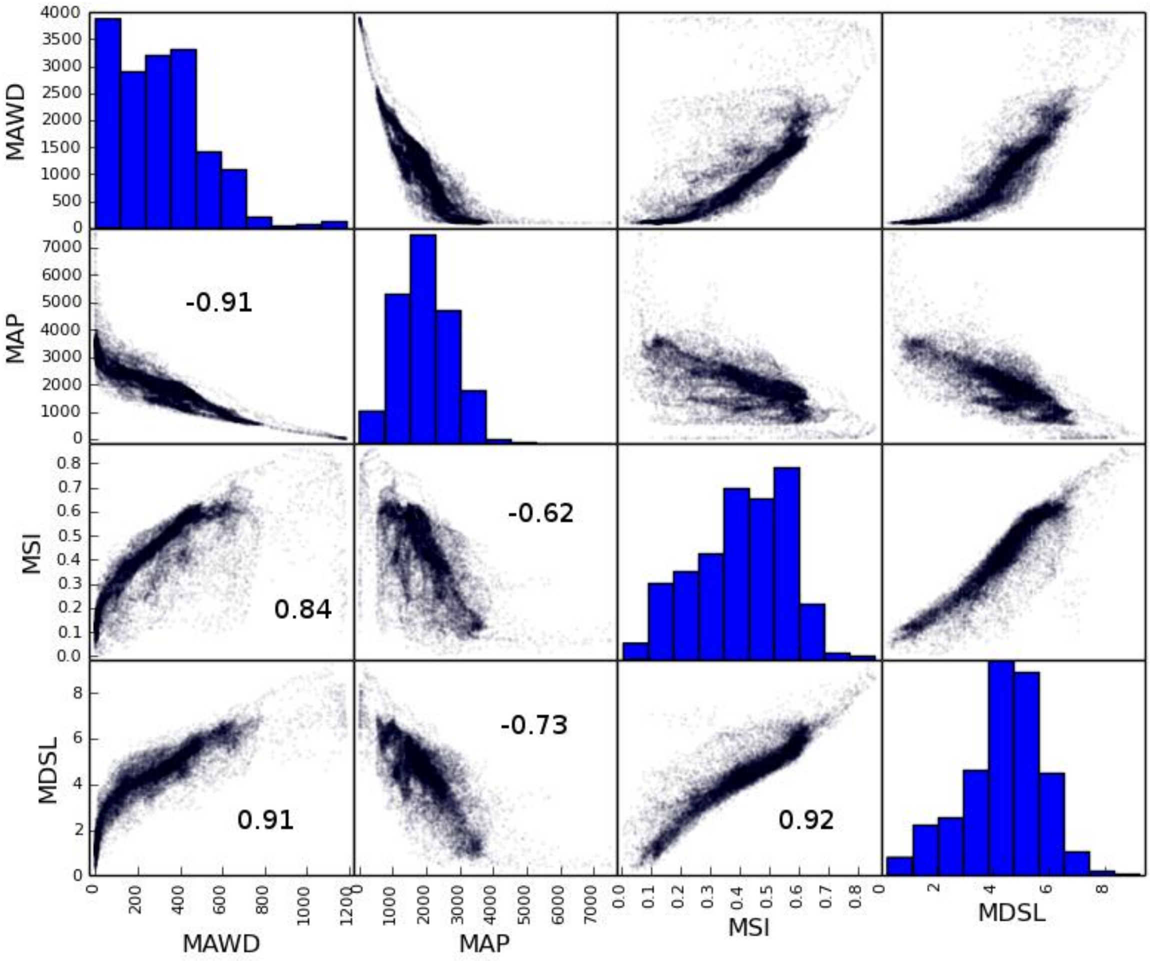

Supplementary Figure 8

Scatterplot matrix of several previously used variables: Mean annual water deficit (MAWD, mean of total annual water deficits), mean annual rainfall (MAP), Markham's seasonality index (MSI) and mean dry season length (MDSL) with histograms and Spearman correlation coefficient. MDSL was calculated by counting the number of months with ≤ 5% of annual rainfall for each year between 1998 and 2010, and then averaging for every location.

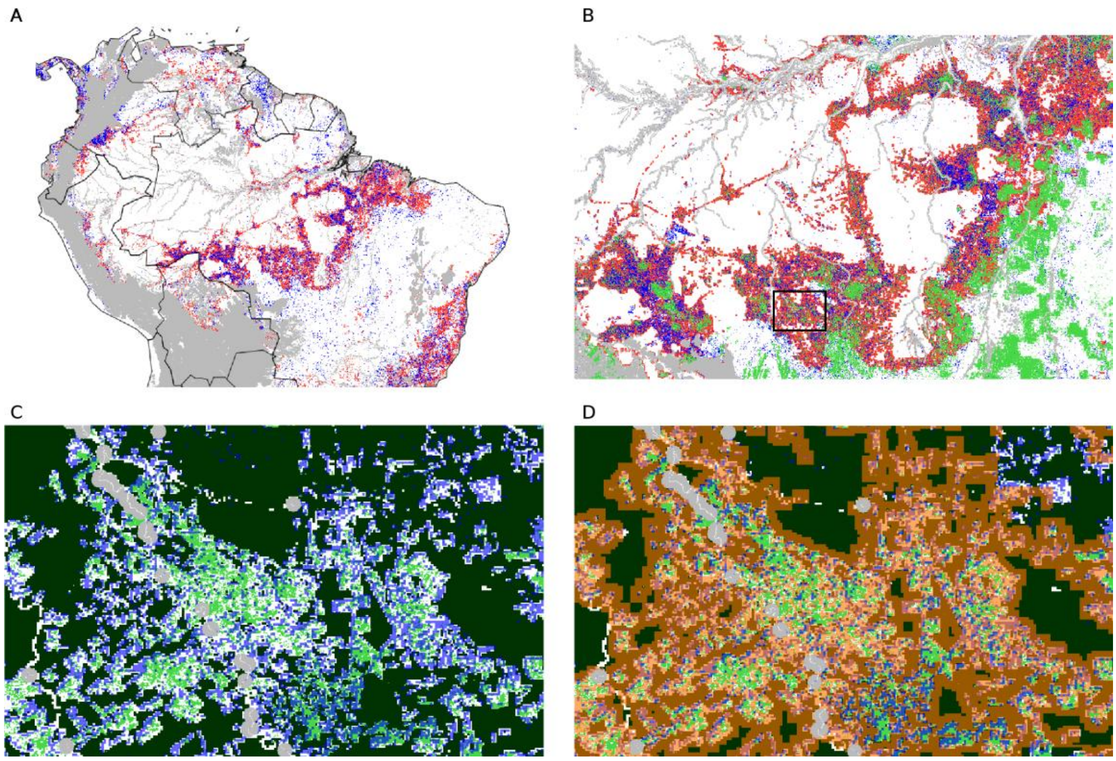

Supplementary Figure 9

Degraded forests and transition areas. (**A**) Blue: areas with degraded forests, defined here as places with tree cover[2] lower than 40% and canopy height[5] greater than 15m. Red: transition areas in places that were forest before the arrivel of man. (**B**) A closer look at a sub-region of (A) with agricultural areas in lightgreen. (**C,D**) A closer look at the sub-region indicated by the rectangle in (B). (**C**) Dark green: forested areas (tree cover >40%). Light green: agricultural areas. Transparent blue: degraded forests. (**D**) As in (C) but with transition areas added as a top transparent orange layer. In all sub-figures, grey areas are the ones that are excluded.

Supplementary Table 2 Model functions. *We model grass cover growth as being only a funciton of rainfall ($k_{g_{M,G}} = 0$). **Only for forest cover *F*, we have taken into account the effect of soil on mortality (m_F). Hence, for all other cover types, $k_{m_{\pi,i}} = 0$.

Name	Function
growth rate	$g_i(M,P) = g_{0,i}\{1\text{-}\exp[k_{g_{M,i}}(M - M_{c_g,i})\text{-}k_{g_{P,i}}(P - P_{c_g,i})]\}$
mortality rate	$m_i(M,P,\pi) = m_{0,i} + \exp[-k_{m_{P,i}}P + k_{m_{M,i}}M - k_{m_{\pi,i}}\pi]^{**}$
savanna tree recruitment rate	$q(\Phi) = q_0(1 - d\Phi)$
soil suitability for forest	$\pi = \theta_1\varphi_s + \theta_2\varphi_c + \theta_3\rho + \theta_4\varphi_c\rho - \pi_c$ (see Table 1)
fire occurrence	$f(M,P,\pi,G+S)$ r.v. drawn from Bernoulli(p) $p = p(M,P,\pi,G+S) = \frac{1}{I}\frac{1}{1+\exp\{-k_f[G+S-C_c(M,P,\pi)]\}}$ with $C_c(M,P,\pi) = 1 - \frac{1-C_0}{1+\exp[-k_M(M-M_0)+k_P(P-P_0)+k_\pi(\pi-\pi_0)]}$
forest cover removal rate	$r(\delta) = r_0\exp(-k_r\delta)$

Supplementary Table 3 Model parameters. S: savanna sapling cover, T: savanna adult tree cover, F: forest tree cover, G: grass cover, M: MSI, P:MAR. Where values of several cover types are given, the order is S,T /F/G, where the comma indicates that the value for S and T are the same. Note that $g_{0,T}$ does not exist and that $m_{0,G} = 0$.

parameter	interpretation	value	unit
$g_{0,S}, g_{0,F}, g_{0,G}$	intrinsic growth rate under favourable conditions	.09/.20/.25	y^{-1}
$m_{0,S}, m_{0,F}, m_{0,G}$	intrinsic mortality rate under favourable conditions	.023/.041/0	y^{-1}
$k_{g_{M,S}}, k_{g_{M,F}}$	rate of decay of intrinsic growth rate with *M*	5/3.26	y^{-1}
$k_{g_{P,S}}, k_{g_{P,F}}, k_{g_{P,G}}$	rate of increase of intrinsic growth rate with *P*	.005/.003/.003	mm^{-1}
$k_{m_{M,F}}$	rate of increase of intrinsic mortality rate with *M*	4.66	-
$k_{m_{P,S}}, k_{m_{P,F}}, k_{m_{P,G}}$	rate of decay of intrinsic mortality rate with *P*	.008/.008/.01	mm^{-1}
$M_{c_g,s}, M_{c_g,F}$	value of *M* where intrinsic growth rate increases sharply	.2/.4	-
$P_{c_g,s}, P_{c_g,F}, P_{c_g,G}$	value of *P* where intrinsic growth rate increases sharply	50/500/0	mm
q_0	*T* recruitment rate without fire	.04	-
d	reduction of *T* recruitment rate in burned areas	0.85	-
b	mortality rate of *F* due to fire in burned areas	0.48	y^{-1}
I	minimum fire return interval	2.75	y
k_f	sharpness of fire probability decrease around $G+S=C_c$	15	-
C_0	min $G+S$ cover threshold beyond which fire spreads	45	%
K_M, K_P, K_π	sensitivity of C_c to MSI/MAR/soil	2.62/.001/.525	
M_0, P_0, π_0	center point for MSI, soil, MAR wrt their effect on C_c	.3/2500/-7.55	-/mm
D	fire diffusion constant	.1	$km^2\ y^{-1}$
r_0	logging rate at the forest edge	.115	y^{-1}
k_r	decay of logging rate with distance from the forest edge	.0015	m^{-1}
σ	standard deviation of the noise	2	-
π_c	center point for soil favourability π	-7.25	-

Supplementary references

1 Libonati, R., DaCamara, C. C., Setzer, A. W., Morelli, F. & Melchiori, A. E. An Algorithm for Burned Area Detection in the Brazilian Cerrado Using 4 µm MODIS Imagery. *Remote Sensing* **7**, 15782-15803 (2015).

2 Townshend, J. *et al.*, Vegetation Continuous Fields MOD44B: 2000-2014 percent tree and herbaceous cover, USGS Earth Resources Observation and Science (EROS) Center, (2011) - lpdaac.usgs.gov/dataset_discovery/modis/modis_products_table/mod44b [21/03/2017]

3 Huffman, G. J., E.F. Stocker, D.T. Bolvin, E.J. Nelkin, R.F. Adler, TRMM 3B43 Version 7, NASA/GSFC, Greenbelt, MD (2012) - trmm.gsfc.nasa.gov [21/03/2017]

4 Phillips, O. L. et al. Drought sensitivity of the Amazon rainforest. *Science* **323**, 1344-1347 (2009).

5 Xu, C. *et al.* Remotely sensed canopy height reveals three pantropical ecosystem states. *Ecology* **97**, 2518–2521 (2016).
